# Clinical, virological and immunological features of HIV-positive children internationally adopted in France from 2005-2015

**DOI:** 10.1371/journal.pone.0203438

**Published:** 2018-09-28

**Authors:** Violaine Corbin, Pierre Frange, Florence Veber, Stéphane Blanche, Camille Runel-Belliard, Muriel Lalande, Virginie Gandemer, Marie Moukagni-Pelzer, Catherine Dollfus, Dilek Coban, Justine Prouteau, Christine Jacomet, Olivier Lesens, for the HIV adopted children study group

**Affiliations:** 1 CHU Clermont-Ferrand, Service des Maladies Infectieuses et Tropicales, Clermont-Ferrand, France; 2 Unité d'Immunologie, Hématologie & Rhumatologie Pédiatriques, AP-HP, Hôpital Universitaire Necker-Enfants Malades, Paris, France; 3 Laboratoire de Microbiologie clinique, AP-HP, Hôpital Universitaire Necker–Enfants Malades, Paris, France; 4 EA7327, Université Paris Descartes, Sorbonne Paris Cité, Paris, France; 5 CHU de Bordeaux, Hôpital Pellegrin, Service des Urgences Pédiatriques, Centre de Competence de la Drépanocytose, Bordeaux, France; 6 Service de Pédiatrie Générale, Infectiologie et Immunologie Clinique, Pôle Femme-Mère-Enfant, CHRU de Montpellier, Hôpital Arnaud de Villeneuve, Montpellier, France; 7 Pôle Femme-Enfant, Service d'Hémato-Oncologie Pédiatrique, CHU Hôpital Sud, Rennes, France; 8 Clinique Pédiatrique Saint Antoine, Hôpital Saint Vincent de Paul, Groupement des Hôpitaux de l’Institut Catholique de Lille (GH-ICL) Lille, France; 9 AP-HP, Service d'Hématologie et d'Oncologie Pédiatrique, Hôpital d'Enfants Armand Trousseau, Paris, France; 10 Laboratoire Microorganismes: Génome Environnement (LMGE) UMR 6023, Université d’Auvergne, Clermont-Ferrand, France; Chiang Mai University, THAILAND

## Abstract

**Objective(s):**

To describe the clinical, virological and immune characteristics of internationally adopted children on arrival in France and after 6-months follow-up.

**Design:**

Multicenter retrospective study.

**Methods:**

30 centers from 24 cities were asked to include, after informed consent, HIV+ children living in France and internationally adopted between 1^st^ Jan 2005 and 1^st^ Jan 2015. Sociodemographic, medical and biological variables collected during the first medical evaluation in France and 6 months later were analyzed.

**Results:**

41 HIV+ adoptees were included (female: 56%; median age: 3.91 years) in 14 centers. Adoptees tend to represent an increasing part of newly diagnosed HIV positive children over the years. The majority came from East-Asia. At arrival, one child was diagnosed with lymphobronchial tuberculosis and three with latent chronic hepatitis B, cleared HBV infection and chronic active hepatitis C, respectively. The mean CD4% was 32.8 ± 9% (range: 13–49%). The 34 children (83%) have been initiated on treatment from their countries of origin. Of these, 25 (74%) had an undetectable viral load (VL) on arrival. Resistance to ART was detected in five. At 6 months, 36 adoptees received ART, and the VL was undetectable in 29 children (71%), with one acquired resistance to NRTI & NNRTI.

**Conclusions:**

An increasing number of HIV-infected children have been internationally adopted in France since 2005. Most of the children have been initiated on treatment from their countries of origin, had good immunity, with few opportunistic infections, and infrequently detectable VL. Low level of mutation conferring resistance was detected.

## Introduction

Some 2.6 million children were living with HIV in the world at the end of 2015 [1]. Most pediatric HIV-1 infections occur in resource-limited countries, mainly in sub-Saharan Africa [1–2]. Immediate initiation of antiretroviral therapy (ART) for all HIV1–infected children is now recommended, and has considerably improved the short- and long-term prognosis of HIV infection, even in countries with limited resources [2]. In perinatally HIV-infected adolescents, the clinical, immunological and virological outcome of ART is good, especially in well-resourced countries with high quality health care access [3]. However, the rate of development of triple-class virological failure in children with HIV remains higher than in adults in Europe, and viral suppression rates among children receiving ART in low- and middle-income countries are lower than in well-resourced countries [4,5]. The ART era has made possible the adoption of HIV-infected children originating from low-resource countries. Since 2004, this possibility has been strengthened by the imbalance between the high numbers of parents applying for adoption, and the lower number of children available for international adoption [6]. As a consequence, parents tend to apply for special-needs children, meaning children older than 5 years, siblings and children with affections diagnosed in the country of origin such as congenital malformations, chronic diseases or developmental disabilities who will require a specific management and follow-up in their adoptive country [7].

In France, the laws of 5 July 1996 and 4 July 2005 govern the adoption, as well as the decree of 1 August 2006 for international adoption. An agreement, delivered by the child welfare services, is required for every application. The procedure depends also on the regulations in the country of origin and/or on the application of the Hague Convention of 29 May 1993 on Protection of Children and Co-operation in respect of Intercountry Adoption. Families can specify their motivation and their needs and whether they would be able or not to manage the adoption of a child with a disability or a chronic disease. 66% of the 815 children adopted in 2015 had special needs, including 25% with pathologies such as chronic infectious diseases [8]. To detect diseases in new-arrival internationally adopted children, 27 adoption clinics (ACs) have been created in France [9]. Routine HIV testing is recommended in the medical evaluation, but the discovery of an HIV-positive test at the time of arrival in the adoptive country is a very rare event [9–11].

About 1,500 HIV-positive children are receiving care in France [12]. Among them, the number of adopted children is unknown and no data are available on the clinical, immunological and viral outcomes of these internationally adopted children. We therefore conducted a multicentric observational study to assess the number of new HIV-infected adoptees in France during year 2005–2015 and to obtain a better description of this specific population.

## Patients & methods

We conducted a retrospective multicenter study in France. Of 17 tertiary care hospitals and 16 adoption clinics (ACs) contacted, 15 hospitals and 15 ACs from 24 cities agreed to participate. Children living in France and internationally adopted between January 1st 2005 and January 1st 2015 were included after written information and written consent was obtained from their adoptive parents. HIV-positive children adopted by a family member (intrafamilial adoption) were not included. Data about adoptive families (including age of the parents and socio-professional background) as well as sociodemographic (type of adoption, date of birth, age on arrival), medical and biological variables regarding adoptees were collected during the first medical evaluation in France, and 6 months later. These variables included the type of HIV, viral load, CD4 cell count, ART and resistance testing). Other data included body mass index (BMI) and nutritional status, the presence of comorbidities and infections (as well as the vaccine status), and the presence or absence of a language delay, defined as a failure to develop language abilities on the usual age-appropriate for the developmental timetable of children. Compliance with treatment was also recorded, defined by difficulties or not for children to take their pills, which may have consequences on the regular intake of ART as prescribed by the physician. In addition, participating centers were asked to indicate the number of new adoptees each year (since 2005) for ACs and the number of new diagnosed HIV children for pediatric departments. The yearly percentage of HIV-positive adoptees was calculated among new adoptees and new HIV-positive children diagnosed. The study was submitted to an IRB, Institutional Review Board (which was the CPP Sud Est VI, Comité de Protection des Personnes) and to other French Ethic Authorities (CCTIRS, Comité consultatif sur le traitement de l'information en matière de recherche dans le domaine de la santé and CNIL, Commission Nationale de l'Informatique et des Libertés). This study was approved by the IRB, the CCTIRS and CNIL before the beginning of the study. The authors had access to any identifying patient information.

## Results

### Incidence

**Fig 1:**
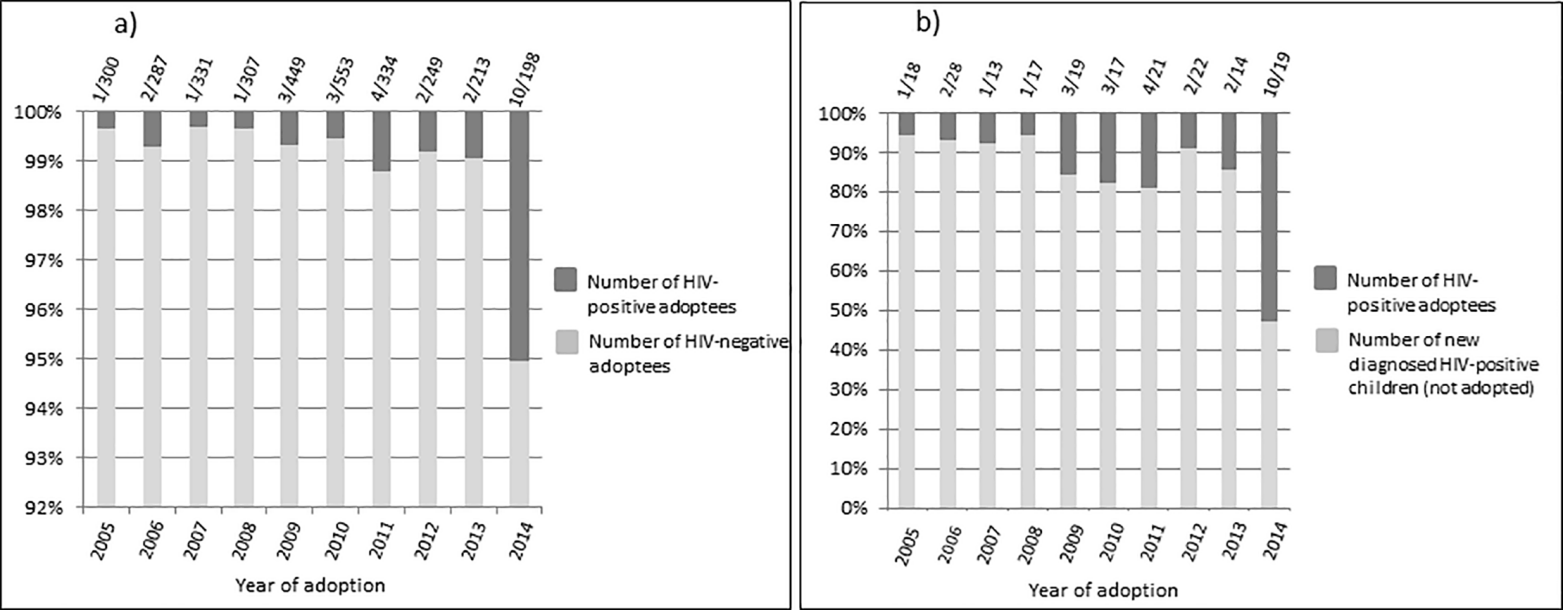
Numbers of HIV-positive adoptees compared with HIV-negative adoptees attending the Centers for Orientation and Counsel in Adoption **(a) and with** the numbers of newly diagnosed HIV-positive children (not adopted) **(b)** in 14 cities giving care to at least one HIV-positive adoptee between 2005 and 2014.

Of the 24 cities participating in the study, 14 gave care to at least one HIV-positive adoptee. 45 HIV-positive children were adopted in these 14 centers between 2005 and 2014. Data of 41 adoptees included in the study (informed consent signed by holders of parental authority) were collected and analyzed. The number of HIV-positive adoptees for each center between 2005 and 2014 was: Paris-Necker Hospital (16), Bordeaux (5), Lille (4), Rennes (4), Angers (3), Clermont-Ferrand (3), Montpellier (2), Paris-Trousseau Hospital (2), Besançon (1), Brest (1), Chambéry (1), Dijon (1), Nice (1), Saint-Nazaire (1). These centers answered the questionnaire about the yearly number of new adoptees and new HIV-positive children. The yearly percentages of HIV-positive adoptees among new adoptees and new HIV-positive children are given in Fig 1A and 1B.

### First medical consultation in France

#### Adoptive families characteristics

87.8% of adopting families were couples, the rest were single mothers (5). 19.5% of adopting fathers and 36.6% of mothers were employees or workers, 43.9% and 24.4% had a managerial position, respectively. There were no retired person. Two couples were farmers. Other categories were liberal profession or craftsperson (9.7% and 14.6%, respectively) and three adopting mothers had no professional activity. 56% of families had at least one other child.

#### Clinical features of children

The first medical consultation was performed by a hospital pediatric department (55%), adoption clinics (ACs, 32.5%), general practitioner (7.5%) or private pediatrician (5%). All the children were known to be HIV-positive before arrival in their adoptive country, except for one 2.5-year-old child who was diagnosed 2 months after arrival in France at the first attendance at the AC. 41 children (23 girls and 18 boys) with a median age of 3.91 years (8 months to 13 years) were adopted. Two children had a history of opportunistic infection (OI) in their country of origin: one child had a recurrent pneumonia classified category C according to the CDC classification and another 6-year-old child from Vietnam had chronic diarrhea classified as category B (moderately symptomatic) [13]. This second child was diagnosed on arrival with lymphobronchial tuberculosis thanks to sputum smear microscopy. Two other children had an active tuberculosis (one still being treated on arrival). Other previously known handicaps were cleft lip and palate, and delayed language acquisition. For three children, a new diagnosis of latent chronic hepatitis B, cleared HBV infection and chronic active hepatitis C was made on arrival in France. Other clinical diagnoses made at the first consultation were benign diseases, mainly skin diseases (Table 1).

**Table 1:** Characteristics of the 41 adoptive families and adoptees.

Adoptive family characteristics	
Couple	87.8% (36)
Mother mean age (years) ± SD^a^ [ranges]	43.4 ± 6 [31–57]
	43.3 ± 5 [33–60]
Father mean age (years) ± SD^a^ [ranges]	56% (23)
	63.4.2% (26)
Family with at least one other child	26.4% (11)
	2.4% (1)
**Countries of origin**	7.4% (3)
East Asia	60% (24)
Africa	2.4% (1)
Haiti	2.4% (1)
East Europe and Russia	34.1% (14)
**Vaccination card from country of origin available**	61% (25)
**Serology available**	2.4% (1/40)
***Hepatitis B***	61% (25/40)
Chronic latent	41.5% (17/21)
Cleared	24.4% (10/12)
Vaccinated	31.7% (13/20)
Negative serology	31.7% (13/20)
***Hepatitis C***	9.7% (4/8)
Chronic active	32% (13)
Negative serology	12.2% (5)
**Proportion of children with positive serology**	14.6% (6)
*Tetanus*	26.8% (11)
*Poliomyelitis*	36.6% (15)
*Diphtheria*	2.4% (1)
*Measles*	
*Mumps*	
**Nutritional status at the first medical consultation in France**	
Body Mass Index <5^th^ percentile	
Malnutrition^b^	
Diarrhea^c^	
Skin disease^d^	
Tuberculosis	
Screening for latent tuberculosis^e^	
Pulmonary tuberculosis	

^a^SD standard deviation

^b^Clinical diagnosis associating a Body Mass Index <5^th^ percentile with one or more clinical signs of malnutrition: attention deficit, dry skin, depigmentation hair changes (hair loss, changing color), muscle wasting, swelling of the abdomen and legs, stick like limbs, spoon-shaped, brittle, ridged nails

^c^Origin: parasite: 2; unknown: 4.

^d^Scabies: 5; tinea: 3; *Molluscum contagiosum*: 2; atopic dermatitis: 1; intertrigo: 1

^e^ Positive tuberculosis skin test or positive Interferon Gamma Release Assay

#### Immune and viral status and antiretroviral therapy

**Fig 2:**
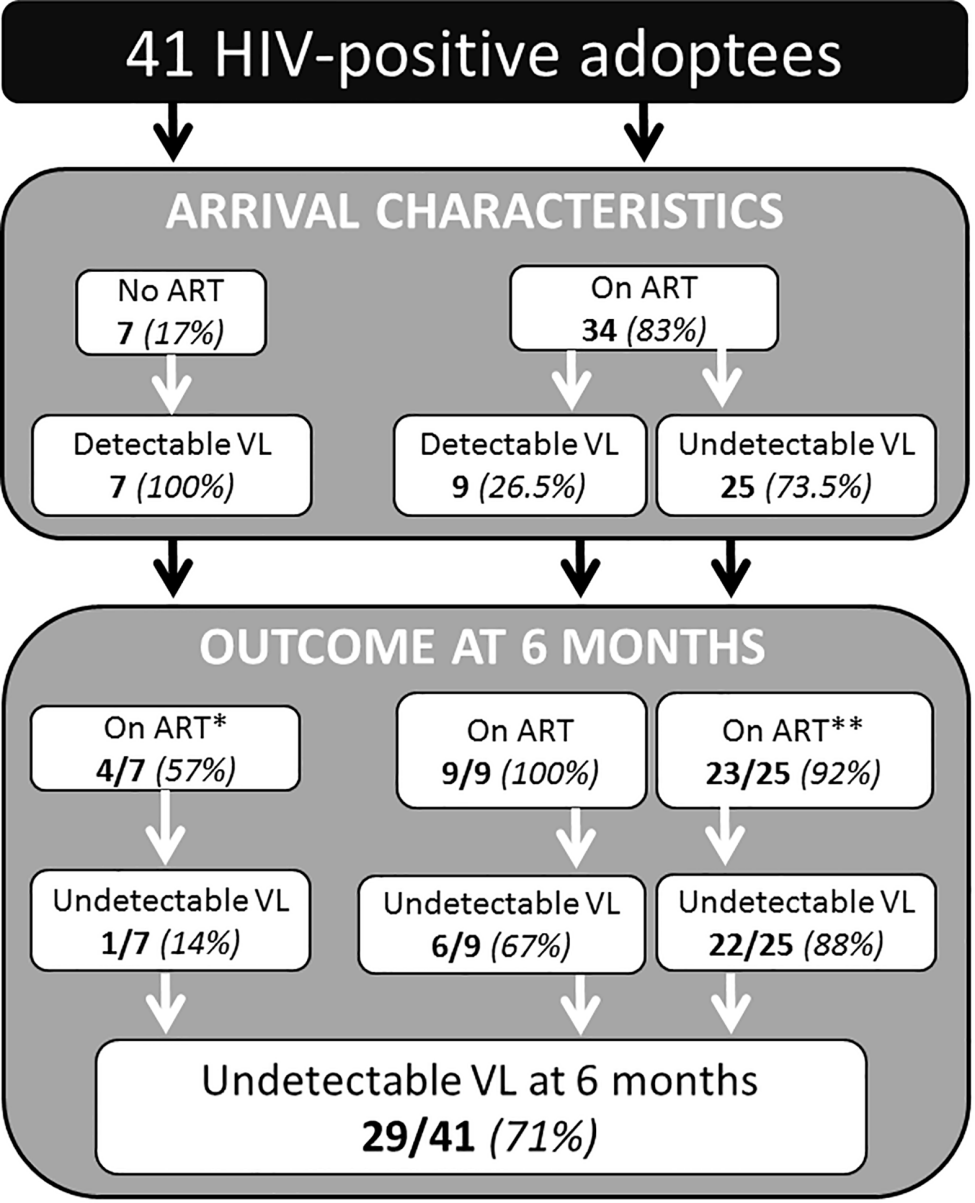
Outcome at 6 months of 41 HIV-positive adoptees (including 36 on ART during the follow-up period). *Three children were not treated at 6 months, one because of tuberculosis; two arrived in 2009 and 2010 respectively and were treated 4 years after arrival. **Two stopped therapy (one because of misunderstanding with the adoptive family and one during period of genotype resistance performance, ART being restarted after 6 months).

All viruses were identified as HIV-1. The mean CD4% was 32.8 ± 9% (ranges: 13–49%). Only one child had a CD4% below 15%. Seven children were receiving cotrimoxazol as a prophylactic therapy. Of the 34 children who received ART in their country of origin, 22 (65%) were treated with two nucleoside/nucleotide reverse transcriptase inhibitors (NRTIs) + one non-nucleoside reverse transcriptase inhibitor (NNRTI), and 11 (32%) with two NRTIs + 1 protease inhibitor (PI). The ART used was unknown for one child (3%). This ART was the second-line treatment for seven children (21%). Of these 34 ART-experienced children, 25 (74%) had an undetectable viral load (VL) on arrival. Among the nine with detectable VL, five had VL under 100 copies/mL, including two for whom nucleic amplification failed owing to low level viremia (Fig 2). One had a VL > 100,000 copies/mL (Child 5, Table 2). Adoptees originating from Southeast Asia were more likely to have an undetectable VL on arrival than those from other countries (p = 0.02). Resistance to ART was detected in five (Table 2) with resistance to NRTIs in four (12%) and resistance to protease inhibitors (PIs) in one child (2.4%). No data were available for genotypic resistance testing in the seven naive children. Of them, five started ART on arrival in France (four children: two NRTIs + one PI; one child: two NRTIs + one NNRTI).

**Table 2:** History of five children with mutations conferring resistance to ART on arrival in France.

Variable	Child 1	Child 2	Child 3	Child 4	Child 5
**Country of birth**	Ethiopia	Vietnam	Russia	Vietnam	Morocco
**Age at start of ART**	2 years	7 months	4 years	3 years	Unknown
**First line therapy**	AZT 3TC NVP	AZT 3TC NVP	Unknown	AZT 3TC EFV	AZT 3TC EFV
**Number of ART lines before arrival in France**	1	1	> 2	1	Unknown
**Age at first evaluation in France**	7 y	5 y	3 y	5 y	3 y
**ART at first evaluation**	AZT 3TC NVP	No therapy	DDI ABC LPV/r	AZT 3TC EFV	AZT 3TC EFV
**VL (copies/ml) at first evaluation**	49	-	720	7000	114 000
**Mutations conferring resistance to NNRTIs at first evaluation**	M184V K103N H221Y	K103N	69N 75T	M184V K219N V90I K101E Y181C G190A	69N 70R 184V K103N
**Mutations conferring resistance to PIs at first evaluation**	None	None	36I 46I 71T 90M	None	None
**ART introduced in France**	TDF 3TC ETR	3TC ABC ETR*	ATC ABC DRV/r	AZT ABC LPV/r	AZT 3TC DRV/r
**VL (copies/ml) at second evaluation**	< 40	18682	<20	<40	388

ART: AntiRetroviral Therapy; VL : Viral Load; y : year; AZT: zidovudin; 3TC: lamivudin; NVP: nevirapin; TDF: tenofovir; ETR: etravirin; ABC: abacavir; DDI: didanosin; LPV/r: lopinavir/rotonavir; DRV: darunavir/ritonavir; EFV: efavirenz.

*ART initiated after second evaluation.

Among the 34 children receiving ART on arrival, 24 continued with the same ART, two stopped therapy (one because of a misunderstanding with the adoptive family, and one during the genotype resistance period, ART being restarted after 6 months), and eight had their ART changed (reasons: one interaction with TB treatments, three therapy failures with viral resistance, one therapy adaptation according to the French protocol and three for treatment intolerance). Finally, among the 36 children who received ART after the first consultation, 20 (56%) received two NRTIs + one NNRTI, and 16 (44%) received two NRTIs + one PI.

Three children were not receiving ART on arrival. One arrived in 2007 (VL: 360,000; CD4: 21%). He was treated for active tuberculosis and started ART in 2008. Two arrived in 2009 and 2010 respectively and were treated only 4 years after arrival: one child because of reluctance from the mother and the other in respect with recommendations on ART of children HIV infection at the time of the 6 months of follow-up.

### Outcome at 6 months

Seven out of 13 children with a *z* score of body mass index (zBMI)[14] between −2 and −3 at arrival were still underweight at 6 months, but the mean gain of zBMI at 6 months was 0.27 (p = 0.043). Between the two consultations, five children were hospitalized for various medical problems: pneumonia, pyelonephritis, ankle cellulitis, tuberculous screening and allergic rash. Of the 32 schooled children, three (two were 5½ years old and one was 7) had fallen behind at school due to problems of adoptive language acquisition.

The medication intake was described as easy and spontaneous by about 90% of the adoptive parents. The VL was undetectable in 29/41 (71%) children. The VL of 7/36 patients (19%) who received ART during the 6 months of follow-up remains detectable (Fig 2). It was <1,000 copies/mL in seven, between 1,000 and 20,000 in two and >100,000 in three. Of them, one child native of Thailand and under 3TC+AZT+LPV/r on arrival acquired resistance to NRTI (M184V) and NNRTI (K103N) after the ART was changed to 3TC+AZT+NVP because of intolerance to LPV/r. These mutations were suspected of being preexistent and revealed by the ART switch. No mutation conferring resistance was found in the remaining six children receiving ART and still detectable at 6 months. The mean CD4% was 35.6 ± 8% (range: 13–49%). The child who was treated for active tuberculosis on arrival had a CD4% below 15% (13%).

## Discussion

This is the first multicenter study focusing on HIV-positive adoptees in France. First we show that an increasing number of HIV-infected children have been internationally adopted in France since 2005. In this study, HIV-positive adoptees represented about half of newly diagnosed HIV-positive children in 2014 versus fewer than 20% in the preceding years. They also represented about 5% of new adoptees in 2014 versus about 1% in the previous years. Among the 41 adoptees included in the study, only one was found seropositive on arrival, stressing the importance of HIV screening for all internationally adopted children (like all migrant children) even if the child had been tested negative in their country of origin. Other adoptees (40/41) were known to be HIV-infected in their country of origin, meaning that the adoptive families had intended specifically to adopt an HIV-positive child. Most of these adoptive families were active middle-class couples who had already experienced adoption or biological filiation. For a number of reasons (the Hague Convention on Protection of Children and Cooperation in Respect of Intercountry Adoption, the emphasis on domestic adoption in many low-income countries and stricter criteria for prospective adoptive parents), HIV-positive children will likely comprise a larger percentage of the children eligible for international adoption. Many adoptive parents may finally choose to adopt an HIV-positive child even though this was not their initial project. This would imply a high level of information, preparation and prolonged support from health care providers to face a difficult challenge that combines the management of adoption and chronic illness. Educational delays were rare in our cohort (9%). This contrasts with the recent study conducted in the US which showed that educational delays were common (50%) [15]. Our second finding is that the majority of HIV-infected adoptees came from East Asia. By contrast, a recent study including HIV-infected children aged <20 years, internationally adopted or in refugee foster care and receiving care in two US hospitals (in Seattle and Denver) from 2004 to 2013 showed that 90% of children came from Africa, mainly Ethiopia [16]. Our third finding was that most children had received ART in their countries of origin, with a majority of undetectable VL on arrival (74% for treated adoptees), good immunity and little resistance to ART. Moreover, Southeast Asian children were more likely to have an undetectable VL than those originating from other countries. In a review focusing on HIV-1 drug resistance after failure of first-line pediatric regimens in children aged <18 years in resource-poor regions, the prevalence of resistance-associated mutations was 90%, including 80% for NRTIs, 88% for NNRTIs and 54% for PIs [15]. In this last study, the prevalence of mutations conferring resistance to multiple NRTIs was high in Asia, possibly because of high exposure to mono- or dual therapy [15]. However, the authors concluded that the drug resistance rates were similar to those reported for children living in Europe [15]. The relatively low prevalence of ART resistance in our cohort may be explained by a better management of HIV infection in adoptees, especially for those coming from Southeast Asia. However, we cannot rule out the possibility of resistance-associated mutations (RAMs) in adoptees with suppressed viremia under ART on arrival, but previously exposed to NNRTI regimens [17].

OIs were rare before or after arrival, as also was hepatitis coinfection (one chronic hepatitis B and one chronic hepatitis C). One new diagnosis of tuberculosis was made after arrival. However, screening for latent tuberculosis was not thorough (36%). Other affections were those classically diagnosed in internationally adopted children, mainly benign skin diseases and diarrhea [7]. Many children were underweight, and malnutrition was diagnosed in five children on arrival. However, the weight gain at 6 months was good.

*Conclusions*: An increasing number of HIV-infected children have been internationally adopted in France since 2005. Most of the children were treated in their country of origin with few OIs and good immunity. VL was frequently undetectable on arrival for adoptees treated with ART in their country of origin with a low level of mutations conferring resistance. Hence the long-term prognosis of these children is likely to be good, but support will be required to face the twofold challenge of adoption management and HIV disease. Future research on the long term outcomes of adopted children living with HIV are needed, especially on virological outcomes but also on behavioral and mental health.

## Supporting information

S1 File Full dataset. (XLSX)
