# Correction: Clinical, virological and immunological features of HIV-positive children internationally adopted in France from 2005-2015

**DOI:** 10.1371/journal.pone.0210933

**Published:** 2019-01-14

**Authors:** The PLOS ONE Staff

In Table 1, the percentages in the right column have been incorrectly shifted up, without spaces between the different categories. Please see the correct Table 1 here. The publisher apologizes for these errors.

**Table 1:** Characteristics of the 41 adoptive families and adoptees.

Characteristics	% (number)
**Adoptive family characteristics**	
Couple	87.8% (36)
Mother mean age (years)) ± SD^a^ [ranges]	43.4 ± 6 [31–57]
Father mean age (years)) ± SD^a^ [ranges]	43.3 ± 5 [33–60]
Family with at least one other child	56% (23)
**Countries of origin**	
East Asia	63.4.2% (26)
Africa	26.4% (11)
Haiti	2.4% (1)
East Europe and Russia	7.4% (3)
**Vaccination card from country of origin available**	60% (24)
**Serology available**	
***Hepatitis B***	
Chronic latent	2.4% (1)
Cleared	2.4% (1)
Vaccinated	34.1% (14)
Negative serology	61% (25)
***Hepatitis C***	
Chronic active	2.4% (1/40)
Negative serology	61% (25/40)
**Proportion of children with positive serology**	
*Tetanus*	41.5% (17/21)
*Poliomyelitis*	24.4% (10/12)
*Diphtheria*	31.7% (13/20)
*Measles*	31.7% (13/20)
*Mumps*	9.7% (4/8)
**Nutritional status at the first medical consultation in France**	
Body Mass Index <5^th^ percentile	32% (13)
Malnutrition^b^	12.2% (5)
Diarrhea^c^	14.6% (6)
Skin disease^d^	26.8% (11)
Tuberculosis	
Screening for latent tuberculosis^e^	36.6% (15)
Pulmonary tuberculosis	2.4% (1)

^a^SD standard deviation

^b^Clinical diagnosis associating a Body Mass Index <5^th^ percentile with one or more clinical signs of malnutrition: attention deficit, dry skin, depigmentation hair changes (hair loss, changing color), muscle wasting, swelling of the abdomen and legs, stick like limbs, spoon-shaped, brittle, ridged nails

^c^Origin: parasite: 2; unknown: 4.

^d^Scabies: 5; tinea: 3; *Molluscum contagiosum*: 2; atopic dermatitis: 1; intertrigo: 1

^e^ Positive tuberculosis skin test or positive Interferon Gamma Release Assay
